# Usher syndrome in Denmark: mutation spectrum and some clinical observations

**DOI:** 10.1002/mgg3.228

**Published:** 2016-06-28

**Authors:** Shzeena Dad, Nanna Dahl Rendtorff, Lisbeth Tranebjærg, Karen Grønskov, Helena Gásdal Karstensen, Vigdis Brox, Øivind Nilssen, Anne‐Françoise Roux, Thomas Rosenberg, Hanne Jensen, Lisbeth Birk Møller

**Affiliations:** ^1^ Applied Human GeneticsKennedy CenterDepartment of Clinical GeneticsCopenhagen UniversityRigshospitaletGlostrupDenmark; ^2^ Department of Cellular and Molecular MedicineThe Faculty of Health SciencesUniversity of Copenhagen2200CopenhagenDenmark; ^3^ Department of Otorhinolaryngology, Head & Neck Surgery and AudiologyBispebjerg Hospital/RigshospitaletCopenhagenDenmark; ^4^ Department of Medical GeneticsUniversity Hospital of North‐NorwayN‐9038TromsøNorway; ^5^ Department of Clinical Medicine, Medical GeneticsUniversity of TromsøNO‐9037TromsøNorway; ^6^ Laboratoire de Génétique MoléculaireCHU MontpellierMontpellierF‐34000France; ^7^ U827InsermMontpellierF‐34000France; ^8^ The National Eye ClinicThe Kennedy CenterDepartment of OphthalmologyCopenhagen University Hospital2600RigshospitaletGlostrupDenmark; ^9^ Institute of Clinical MedicineThe Faculty of Health SciencesUniversity of Copenhagen2200Copenhagen NDenmark; ^10^ Department of Science Systems and Models (NSM)Roskilde UniversityDK 4000RoskildeDenmark

**Keywords:** Cataract, Denmark, macular edema, mutation, olfactory function, targeted NGS, Usher syndrome

## Abstract

**Background:**

Usher syndrome (USH) is a genetically heterogeneous deafness‐blindness syndrome, divided into three clinical subtypes: USH1, USH2 and USH3.

**Methods:**

Mutations in 21 out of 26 investigated Danish unrelated individuals with USH were identified, using a combination of molecular diagnostic methods.

**Results:**

Before Next Generation Sequencing (NGS) became available mutations in nine individuals (1 USH1, 7 USH2, 1 USH3) were identified by Sanger sequencing of *USH1C*,*USH2A* or *CLRN1* or by Arrayed Primer EXtension (APEX) method. Mutations in 12 individuals (7 USH1, 5 USH2) were found by targeted NGS of ten known USH genes. Five novel pathogenic variants were identified. We combined our data with previously published, and obtained an overview of the USH mutation spectrum in Denmark, including 100 unrelated individuals; 32 with USH1, 67 with USH2, and 1 with USH3. Macular edema was observed in 44 of 117 individuals. Olfactory function was tested in 12 individuals and found to be within normal range in all.

**Conclusion:**

Mutations that lead to USH1 were predominantly identified in *MYO7A* (75%), whereas all mutations in USH2 cases were identified in *USH2A*. The *MYO7A* mutation c.93C>A, p.(Cys31*) accounted for 33% of all USH1 mutations and the *USH2A* c.2299delG, p.(Glu767Serfs*21) variant accounted for 45% of all USH2 mutations in the Danish cohort.

## Introduction

Usher syndrome (USH) is an autosomal recessive deafness‐blindness disorder characterized by congenital hearing impairment, progressive visual loss due to retinitis pigmentosa (RP), and vestibular dysfunction in some cases. The prevalence has been estimated to be 3.5–16.6 in 100,000 (Boughman et al. 1983; Grondahl and Mjoen 1986; Rosenberg et al. 1997; Kimberling et al. 2010). Based on clinical findings USH is divided into three subtypes*;* USH1 (OMIM #276900*)*, USH2 (OMIM #276901) and USH3 (OMIM #276902) differentiated by the degree of hearing loss, the age of onset of RP and the presence or absence of vestibular function (Moller et al. 1989; Smith et al. 1994). USH1 is the most severe form of the three types and is characterized by severe to profound congenital hearing impairment, prepubertal onset of RP, and vestibular dysfunction. USH2 is defined by congenital moderate to severe hearing impairment, onset of RP in the first or second decade of life, and normal vestibular function. USH3 is characterized by a congenital or early onset of progressive hearing impairment, whereas the onset and severity of RP as well as the vestibular function are highly variable (Smith et al. 1994; Hope et al. 1997). Currently, 14 genes associated with USH have been identified. Six genes involved in USH1 have been identified: *MYO7A* (OMIM *276903), *USH1C* (OMIM *605242), *PCDH15* (OMIM *605514), *USH1G* (OMIM *607696), *CDH23* (OMIM *605516), and *CIB2* (OMIM *605564). Three genes underlying USH2 have been identified: *USH2A* (OMIM *608400), *ADGRV1* (OMIM *602851), and *DFNB31* (OMIM *607084). In addition, *CLRN1* (OMIM *606397) has been ascertained in USH3 and both *HARS* (OMIM *614504) and ABHD12 (OMIM *613599) in atypical USH (Joensuu et al. 2001; Ness et al. 2003; Eisenberger et al. 2012; Puffenberger et al. 2012). Finally, two modifier genes, *PDZD7* (OMIM *612971) and CEP250 (OMIM *609689) have been recognized (Ebermann et al. 2010; Khateb et al. 2014). The encoded proteins interact in protein complexes in the hair cells of the inner ear, and in the photoreceptors of the eye (Kremer et al. 2006; Reiners et al. 2006; El‐Amraoui and Petit 2014). Individuals with USH from Denmark have previously been investigated for mutations in USH genes. In an early study individuals with USH1 were investigated for mutations in *MYO7A* (Janecke et al. 1999; Dreyer et al. 2000, 2008) investigated individuals with USH2 for mutations in *USH2A*. Mixed groups of USH cases have been investigated for mutations in several genes using Arrayed Primer EXtension (APEX); Cremers et al. 2007; Tranebjærg et al. 2011. Recently USH patients were investigated for exon deletions/duplications in *USH2A* and *PCDH15* by Multiplex ligation‐dependent probe amplification (MLPA); Dad et al. 2015.

This study presents the results of molecular examinations of 26 individuals with USH residing in Denmark using a combination of APEX microarray for identification of previously reported mutations, traditional Sanger sequencing of single candidate genes, or targeted Next‐Generation Sequencing (NGS) of a panel of 10 genes, associated with USH.

By combining the mutation data obtained in this study with all previously published molecular data on USH patients from Denmark we obtained a overview of the identified mutations in 100 Danish individuals with USH.

## Materials, Subjects and Methods

### Ethical compliance

The study was approved by the Regional Research Ethics Committee (H‐3‐2011‐070) and carried out in accordance with the Declaration of Helsinki.

### Patients

**Figure 1:**
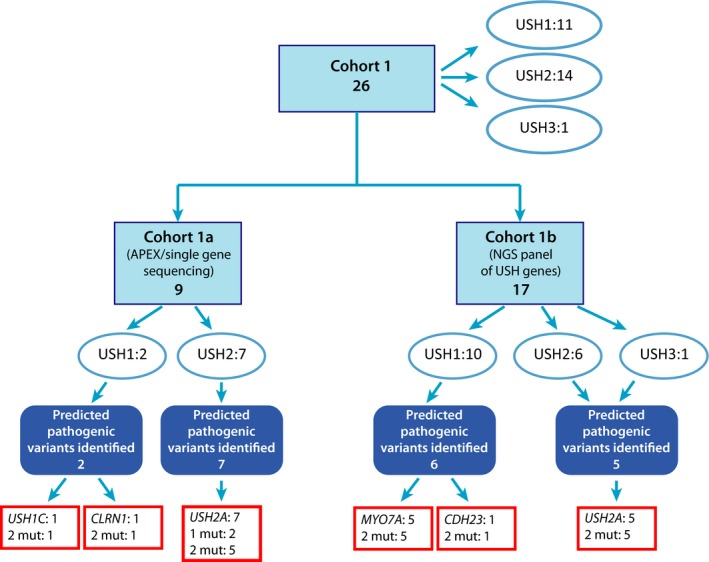
Flow diagram of the mutation screening of cohort 1. The figure shows an overview of the total number of individuals in the cohort 1, which is divided to subcohort 1a and subcohort 1b. Cohort 1 consists of all individuals investigated in this study. Cohort 1a consists individuals investigated by other methods before Next Generation Sequencing (NGS) became available, and cohort 1b consists of individuals analyzed by targeted NGS of known USH genes. Mut = mutation(s).

In this study, 26 unrelated individuals with USH residing in Denmark (cohort 1, Fig. 1) were investigated for mutations.

By combining the mutation data obtained in this study with all previously published molecular data on 118 patients belonging to 100 families with USH registered at the Danish National Eye Clinic for the Visually Impaired at the Kennedy Center (whole cohort), we obtained an overview of all disease causing mutations hitherto identified in 100 Danish individuals with USH. Additional 5 patients with no mutations identified are registered. In addition to the mutations, the gender and some clinical symptoms of the patients are noted in Tables S1–S3.

Consents to genetic testing were obtained for all included individuals.

### Clinical examinations

All patients were diagnosed at the National Eye Clinic (Rigshospitalet, Denmark). Based on routine clinical examinations including visual acuity, slit‐lamp examination, fundoscopy, visual field examination, full‐field electroretinography (ffERG), and optical coherence tomography (OCT). Audiological information from different out‐patient clinics was available from 70 patients from the whole cohort (15 USH1, 54 USH2 and 1 USH3). Vestibular function was not assessed in this study. A majority of the information came from practicing otologists who normally do not obtain vestibular function. Information regarding the onset of visual and hearing impairment was retrospectively sampled from the files of the patients. Supplementary information was received from the patients upon request. Forty‐six individuals (42 families; 13 USH1, 28 USH2 and 1 USH3) completed a questionnaire including general information about onset of night blindness, hearing loss and family history. Despite the missing information on vestibular function our diagnostic classification had a high precision.

### Olfactory function

Olfactory function was tested with the standardized Sniffin’ Sticks 12‐item test (Burghart, Wedel, Germany). The kit includes 12 felt‐tip pens, where each pen contains one common odor (orange, leather, cinnamon, peppermint, banana, lemon, liquorice, coffee, cloves, pineapple, rose and fish). The test comprises a forced choice identification task, where the individual is presented to each pen for 30 sec, and is then asked to select the correct odors from four possible answers. The results generate a score compared to the age of normalized data presented in a normogram. According to the manufactures protocol a score >10 is normal and a score ≤10 suggests that further evaluation should be made. A score of ≤6 indicates severe olfactory dysfunction including anosmia (Hummel et al. 2007).

### DNA extraction

Genomic DNA was extracted from blood leukocytes according to standard procedures.

### Mutation detection methods

- Usher APEX microarray. DNA samples were analyzed by Asper Biotech (Tartu, Estonia) for the presence of known nucleotide substitutions and small deletions/insertions in USH genes using the Usher APEX microarray as described previously (Cremers et al. 2007). Two different versions of APEX were used; version 5 (containing probes for 429 different USH mutations) and version 6 (containing probes for 612 different USH mutations).
- Screening of selected single genes. Coding sequences and flanking intronic sequences of *USH1C*,* USH2A*, and *CLRN1* were analyzed by Sanger sequencing, as described previously (Dreyer et al. 2000, 2008; Roux et al. 2006; Tranebjærg et al. 2011).
- Targeted NGS of Usher genes. DNA samples were analyzed using a customized NGS oligonucleotide‐based target capture USH‐panel (Otogenetics Corporation, Norcross, GA). The USH panel included coding regions and flanking intronic sequences of nine known genes for USH: *MYO7A*,* PCDH15*,* CDH23*,* USH1C*,* USH1G*,* USH2A*,* ADGRV1*,* DFNB31*,* CLRN1*, and the modifier gene PDZD7. Sequencing was carried out using 100 bp paired‐end reads on an Illumina Hiseq2000 sequencer with minimum guaranteed on‐target average coverage of 50–100X. The actual average coverage was very high (450X). Two exons were not covered by NGS; USH2A exon 1 (133 bp) and maximum 72 bp out of 617 bp of DFNB31 exon 9. These two exons were Sanger sequenced. Initial bioinformatics mapping to reference sequence, variant calling and filtering was performed by Otogenetics Corporation, delivered through http://www.dnanexus.com together with a mutation report. Databases, Alamut (Interactive Biosoftware v2.4) and HGMDprof (http://www.biobase-international.com/product/hgmd) were used to classify and annotate the identified variants. Potentially pathogenic variants were verified by Sanger sequencing. Primers used for verification of identified variants are listed along with PCR conditions in Table S4. Primer sequences used for sequencing of uncovered exons are available upon request. We were able to detect homozygous deletions or duplications with the NGS panel data, but have not performed copy number analysis for detection of heterozygous deletions/duplications.
- *USH2A* Individuals with USH2 and no or only one identified mutation were analyzed for the pathogenic intronic variant c.7595‐2144A>G in *USH2A* (Vache et al. 2012) by Sanger sequencing. Primers used for PCR amplification are listed along with PCR conditions in Table S4.

### Accession numbers

Nomenclature of mutations is according to HGVS (www.hgvs.org) and based on the following accession numbers; NM_000260.3 (*MYO7A)*, NM_033056.3 (*PCDH15)*, NM_022124.5 (*CDH23)*, NM_005709.3 (*USH1C)*, NM_173477.4 (*USH1G)*, NM_206933.2 (*USH2A*), NM_032119.3 (*ADGRV1)*, NM_015404.3 (*DFNB31)*, NM_174878.2 (*CLRN1)*, and NM_001195263.1 (*PDZD7)*.

## Results

### Pathogenic variants identified in 21 unrelated USH individuals (cohort 1)

We identified pathogenic variants in 21 out of 26 unrelated individuals affected with USH; cohort 1 (Fig. 1; Table 1).

**Table 1:** Mutations identified in 21 unrelated individuals (cohort 1)

Patient	Gene	Allele 1	Allele 1 Predicted protein	Exon	Allele 2	Allele 2 Predicted protein	Exon	Method
USH1‐2	*MYO7A*	c.93C>A	p.(Cys31*)	3	c.93C>A	p.(Cys31*)	3	A
USH1‐31	*MY07A*	c.93C>A	p.(Cys31*)	3	c.93C>A	p.(Cys31*)	3	A
USH1‐1	*MYO7A*	c.805_807del	p.(Lys269del)	8	c.93C>A	p.(Cys31*)	3	A
USH1‐30	*MYO7A*	c.1708C>T	p.(Arg570*)	15	c.1708C>T	p.(Arg570*)	15	A
USH1‐3	*MYO7A*	c.3503G>A	p.(Arg1168Gln)	27a	c.93C>A	p.(Cys31*)	3	A
USH1‐36	*CDH23*	**c.3862C>T**	p.(Gln1288*)	32a	**c.3862C>T**	p.(Gln1288*)	32a	A
USH1‐6	*CDH23*	**c.6517G>T**	p.(Glu2173*)	48	**c.6517G>T**	p.(Glu2173*)	48	A
USH1‐6B	*CDH23*	**c.6517G>T**	p.(Glu2173*)	48	**c.6517G>T**	p.(Glu2173*)	48	
USH1‐13	*USH1C*	**c.1146dupA**	p.(Gln383Thrfs*6)	14	**c.1146dupA**	p.(Gln383Thrfs*6)	14	C
USH2‐1	*USH2A*	c.1606T>C	p.(Cys536Arg)	9	c.1606T>C	p.(Cys536Arg)	9	A
USH2‐34	*USH2A*	**c.1647T>G**	**p.(Cys549Trp)**	6	c.7524delT	p.(Arg2509Glyfs*19)	40	D
USH2‐3	*USH2A*	c.2299delG	p.(Glu767Serfs*21)	13	c.2299delG	p.(Glu767Serfs*21)	13	A
USH2‐11	*USH2A*	c.2299delG	p.(Glu767Serfs*21)	13	c.4106C>T	p.(Ser1369Leu)	19	B
USH2‐17	*USH2A*	c.2299delG	p.(Glu767Serfs*21)	13	c.2299delG	p.(Glu767Serfs*21)	13	B
USH2‐21	*USH2A*	c.2299delG	p.(Glu767Serfs*21)	13	NI	NI		D
USH2‐59	*USH2A*	c.2299delG	p.(Glu767Serfs*21)	13	c.2299delG	p.(Glu767Serfs*21)	13	
USH2‐52	*USH2A*	c.2299delG	p.(Glu767Serfs*21)	13	c.3407G>A	p.(Ser1136Asn)	17	D
USH2‐52a	*USH2A*	c.2299delG	p.(Glu767Serfs*21)		c.3407G>A	p.(Ser1136Asn)		
USH2‐29	*USH2A*	c.2522C>A	p.(Ser841Tyr)	13	NI	NI		B
USH2‐2	*USH2A*	c.9370A>G)	p.(Arg3124Gly)	47	c.2299delG	p.(Glu767Serfs*21)	13	A
USH2‐9	*USH2A*	c.10561T>C	p.(Trp3521Arg)	53	c.486‐14G>A	p.(?)	Intron 2	A
USH−20^a^	*USH2A*	c.14384T>G	p.(Leu4795Arg)	66	c.2299delG	p.(Glu767Serfs*21)	13	A
USH3‐1^b^	*CLRN1*	**c.254‐1G>A (IVS1−1G>A)**	**p.(?)**	1	**c.254‐1G>A (IVS1−1G>A)**	**p.(?)**	1	E

NI, not identified.

Mutations identified in 21 unrelated individuals (and family members) from a cohort of 26 individuals with USH using different methods. A: targeted NGS of USH genes, B: APEX microarray (Dreyer et al. 2008). C: *USH1C* Sanger sequencing (Dad et al. 2015). D: *USH2A* Sanger sequencing (Janecke et al. 1999; Dreyer et al. 2000). E: *CLRN1* Sanger sequencing (Cremers et al. 2007).

Mutations in **bold** are novel according to HGMDprof and LOVD USH databases 081015.

a Initially diagnosed as USH3.

b Initially diagnosed as USH1. Nomenclature of mutations is based on the following accession numbers; NM_000260.3 (*MYO7A)*, NM_022124.5 (*CDH23)*, NM_005709.3 (*USH1C)*, NM_206933.2 (*USH2A*) and NM_174878.2 (*CLRN1)*. Nomenclature is according to HGVS (www.hgvs.org).

Subcohort 1a: In the period of 2007–2009, before NGS became available, mutations in DNA samples from nine of these individuals (USH1 [*n* = 2], USH2 [*n* = 7]) were identified using a combination of Asper APEX microarray for identification of previously reported USH mutations and Sanger sequencing of *USH2A*,* USH1C* and *CLRN1*. *USH2A* mutations were identified in the seven persons with USH2. Furthermore, for one person with USH1, mutations were identified in *USH1C* (USH1‐13). In another person clinically diagnosed with USH1, mutations were identified in *CLRN1* (USH3‐1). We identified two mutations in seven patients (1 USH1, 5 USH2, 1 USH3) and one mutation in two patients (2 USH2; Table 1; Tables S1–S3).

Subcohort 1b: The remaining 17 individuals were analyzed for mutations by targeted NGS of the genes known to be associated with USH. Two mutations were identified in twelve individuals (USH1 [*n* = 7], USH2 [*n* = 4] and USH3 [*n* = 1]). *MYO7A* mutations were identified in five USH1 persons, *CDH23* mutations in two USH1 individuals, and *USH2A* mutations in five USH2 persons. The person clinically diagnosed with USH3 turned out to have mutations in *USH2A* (USH2‐20). In 5 individuals (3 USH1 and 2 USH2) no pathogenic variants were identified (Fig. 1).

Vache et al. (2012) reported the intronic pathogenic variant c.7595‐2144A>G in *USH2A*, resulting in the insertion of a pseudoexon. This variant is located deep into the intronic sequence and would therefore not be identified by either traditional *USH2A* screening or targeted NGS. We decided to investigate the remaining USH2 individuals, with no or only one identified mutation in *USH2A*, for this variant (e.g. USH2‐21, USH2‐29, USH2‐74 and USH2‐77). However, none of these cases harbored this variant.

In total, two mutations were detected in 19 of the unrelated individuals in cohort 1, and only one mutation was detected in two unrelated individuals; in total, 19 different mutations were detected. The identified mutations and the method used are listed in Table 1.

Five novel mutations were identified: c.6517G>T, p.(Glu2173*) and c.3862C>T, p.(Gln1288*) both in *CDH23* (USH1‐6; USH‐1‐36), c.1146dupA, p.(Gln383fs*6) in *USH1C* (USH1‐13), c.1647T>G, p.(Cys549Trp) in *USH2A* (USH2‐34) and the splice mutation, c.254‐1G>A in *CLRN1* (USH3‐1). Only the c.6517G>T, p.(Glu2173*) mutation in *CDH23* was identified previously and reported in the ExAC database with a frequency of 0.0017%. The other four mutations were not observed in approximately 6500 individuals of European and African American ancestry in the NHLBI Exome Sequencing Project nor in the ExAC database or in Whole‐exome sequencing of 2000 Danish individuals (Lohmueller et al. 2013). No patients with possible digenic inheritance of USH were identified in cohort 1a.

### Overview of mutations identified in the Danish USH population

To obtain an overall picture of molecular findings in patients with USH registered at the Danish National Eye Clinic for the Visually Impaired, we pooled the mutation data obtained from cohort 1 (21 unrelated cases) with all previously published mutation data from Danish USH patients (79 unrelated cases; Janecke et al. 1999; Dreyer et al. 2000, 2008; Cremers et al. 2007; Tranebjærg et al. 2011; Dad et al. 2015). In total, we collected mutation data from 100 USH probands. Table 2 lists all mutations identified in the whole cohort.

**Table 2:** Mutations identified in 100 individuals with USH from Denmark

Gene	Mutation	Predicted protein	Exon	Alleles	Classification
*MYO7A*					
Nonsense mutations	c.93C>A	p.(Cys31*)	3	19	Pathogenic
	c.1708C>T	p.(Arg570*)	15	2	Pathogenic
	c.1996C>T	p.(Arg666*)	17	1	Pathogenic
	c.2055C>A	p.(Tyr685*)	17	1	Pathogenic
	c.5215C>T	p.(Arg1739*)	38	1	Pathogenic
	c.5392C>T	p.(Gln1798*)	39	1	Pathogenic
	c.5824G>T	p.(Gly1942*)	42	1	Pathogenic
Missense mutations	c.634C>T	p.(Arg212Cys)	7	2	Pathogenic
	c.905G>A	p.(Arg302His)	9	1	UV2
	c.3503G>A	p.(Arg1168Gln)	27a	1	UV3
	c.3719G>A	p.(Arg1240Gln)	29	3	Pathogenic
	c.3862G>C	p.(Ala1288Pro)	30	1	UV3
	c.4882G>T	p.(Ala1628Ser)	36	3	UV2
Deletions/duplications/insertions	c.805_807delAAG	p.(Lys269del)	8	1	UV3
	c.3040_3043delinsTACTTCCAGGGGACA	p.(Thr1014Tyrfs*52)	24	2	Pathogenic
	c.6025delG	p.(Ala2009Profs*32)	44	1	Pathogenic
Splice site mutations	c.1555‐8C>G	(IVS13‐8C>G) p.(?)	13	1	UV4
*CDH23*					
Nonsense mutations	c.3862C>T	p.(Gln1288*)	32a	2	Pathogenic
	c.6517G>T	p.(Glu2173*)	48	2	Pathogenic
Splice site mutations	c.4489‐2A>C	p.(?)	35	2	Pathogenic
	c.6050‐9G>A	p.(?)	45	2	Pathogenic
	c.7872G>A	p.(Glu2624Glu)/p.(?)	54	2	UV4
*USH1C*					
Nonsense mutations	c.91C>A	p.(Arg31*)	2	1	Pathogenic
Deletions/duplications/insertions	c.238dup	p.(Arg80Profs*69)	3	3	Pathogenic
	c.1146dup	p.(Gln383fs*6)	14	2	Pathogenic
*USH2A*					
Nonsense mutations	c.187C>T	p.(Arg63*)	2	1	Pathogenic
	c.1876C>T	p.(Arg626*)	11	2	Pathogenic
	c.2023C>T	p.(Gln675*)	12	3	Pathogenic
	c.2028C>A	p.(Cys676*)	12	1	Pathogenic
	c.2797C>T	p.(Gln933*)	13	2	Pathogenic
	c.3309C>A	p.(Tyr1103*)	16	1	Pathogenic
	c.4957C>T	p.(Arg1653*)	24	1	Pathogenic
	c.5473G>T	p.(Glu1825*)	27	1	Pathogenic
	c.5653A>T	p.(Arg1885*)	28	1	Pathogenic
	c.9120G>A	p.(Trp3040*)	46	3	Pathogenic
	c.10684G>T	p.(Glu3562*)	54	2	Pathogenic
	c.11416G>T	p.(Glu3806*)	59	1	Pathogenic
	c.11864G>A	p.(Trp3955*)	59	3	Pathogenic
Missense mutations	c.488G>A	p.(Cys163Tyr)	3	1	UV2
	c.949C>A	p.(Arg317Arg)	6	1	UV4
	c.1000C>T	p.(Arg334Trp)	6	1	Pathogenic
	c.1036A>C	p.(Asn346His)	6	1	Pathogenic
	c.1606T>C	p.(Cys536Arg)	9	4	Pathogenic
	c.1647T>G	p.(Cys549Trp)	10	1	UV3
	c.2137G>C	p.(Gly713Arg)	12	1	UV2
	c.2276G>T	p.(Cys759Phe)	13	2	Pathogenic
	c.2522C>A	p.(Ser841Tyr)	13	1	UV1
	c.3407G>A	p.(Ser1136Asn)	17	1	UV3
	c.3635C>T	p.(Pro1212Leu)	17	1	UV3
	c.4106C>T	p.(Ser1369Leu)	19	1	UV3
	c.5270A>G	p.(Tyr1757Cys)	26	1	UV2
	c.9370A>G	p.(Arg3124Gly)	47	2	UV3
	c.10510C>A	p.(Pro3504Thr)	53	2	UV3
	c.10561T>C	p.(Trp3521Arg)	53	4	UV3
	c.12161G>T	p.(Ser4054Ile)	62	2	UV3
	c.13316C>T	p.(Thr4439Ile)	63	3	UV3
	c.13776G>C	p.(Gln4592His)	63	1	UV3
	c.14384T>G	p.(Leu4795Arg)	66	2	UV3
Deletions/duplications/insertions	c.672_1840+1160del	p.(Ser224Argfs*5)	4‐10	2	Pathogenic
	c.920_923dup	p.(His308Glnfs*16)	6	5	Pathogenic
	c.1965delT	p.(Cys655Trpfs*101)	11	1	Pathogenic
	c.2299delG	p.(Glu767Serfs*21)	13	56	Pathogenic
	c.2878_2879delAA	p.(Asn960Serfs*4)	14	2	Pathogenic
	c.4628_4987del	p.(Gly1543_Pro1662del)	22‐24	1	Pathogenic
	c.6795_6797delATA	p.(Glu2265_Tyr2266delinsAsp)	35	1	UV3
	c.7195_7207del	p.(Ile2399Phefs*10)	38	1	Pathogenic
	c.7524delT	p.(Arg2509Glyfs*19)	40	1	Pathogenic
	c.9770dup	p.(Asn3257Lysfs*9)	50	1	Pathogenic
	c.10345delinsAA	p.(Glu3449Lysfs*25)	52	1	Pathogenic
Splice site mutations	c.486‐14G>A	p.(?)	Intron 2	1	UV4
*CLRN1*					
Splice site mutations	c.254‐1G>A	p.(?)		2	Pathogenic

Mutations identified in 100 individuals with USH from Denmark. The pathogenity is defined according to LOVD Usher database. Classification: Neutral – UV1 (certainly neutral) – UV2 (likely neutral) – UV3 (likely pathogenic) – UV4 (certainly pathogenic) – Pathogenic. Novel nonsense and splice site mutations were classified as pathogenic and prediction of the pathogenity of the novel missense mutations were performed in silico, using the web tools SIFT, Mutation taster, Polyphen 2 and Align GVGD. Nomenclature of mutations is based on the following accession numbers; NM_000260.3 (*MYO7A)*, NM_022124.5 (*CDH23)*, NM_005709.3 (*USH1C)*, NM_206933.2 (*USH2A*) and NM_174878.2 (*CLRN1)*. Nomenclature is according to HGVS (www.hgvs.org).

#### Mutations in patients with USH1

In the combined Danish cohort, one or two mutations were identified in 32 USH1 families (36 individuals; Table 2; Table S1). Mutations were identified in *MYO7A* in 75% (24/32), in *CDH23* in 16% (5/32), and in *USH1C* in 9% (3/32) of the families. No mutation was identified in either *PCDH15* or *USH1G*. Two mutations were identified in 26 persons and one mutation in six.

Nonsense and missense mutations were observed most frequently. Splice site mutations were only identified in *MYO7A* and *CDH23*. The majority of the mutations only appeared in a single patient (in heterozygous or homozygous state). In contrast, the *MYO7A* mutation p.Cys31* had an allele frequency of 45% (19 out of 42 *MYO7A* alleles with identified mutation), and accounted for 33% of all pathogenic USH1 alleles (19/58). The c.238dup mutation in *USH1C* appeared in two patients.

The pathogenic variants in the *CDH23* gene; *c*.3862C>T, p.(Gln1288*), c.4489‐2A>C, c.6050‐9G>A and c.7872G>A, p.(Glu2624Glu), were identified in individuals of Iraqi, Sri Lankan or Pakistani origin (Table S1). Only the mutation c.6517G>T, p.(Glu2173*) was identified in a person with Danish origin (USH1‐6). Two nonsense mutations p.(Gln1288*) and p.(Glu2173*) were identified. The additional three mutations all affect the splicing of the *CDH23* transcript, including the “silent” mutation c.7872G>A, p.(Glu2624Glu) located at the last basepair in exon 54. This mutation is assumed to activate a cryptic splice site in intron 54, leading to extension of exon 54 with 86 nucleotides and resulting in a frame shift (Vache et al. 2012).

While the majority of pathogenic variants in *MYO7A* were identified in individuals with Danish origin, only two pathogenic variants, c.3040_3043delinsTACTTCCAGGGGACA and c.1708C>T, were identified in individuals of Turkish and Sri Lankan origin, respectively.

#### Mutations in patients with USH2

In the combined Danish cohort, 67 unrelated USH2 families (81 individuals) harbored mutations in one or both alleles, all affecting *USH2A* (Table 2; Table S2). Two patients (USH2‐13 and USH‐60) had actually three mutations in total, two on the same allele (Table S2). In this study, the splice variant, c.486‐14G>A in intron 2 in *USH2A*, was identified in trans with a missense mutation c.10561T>C, p.(Trp3521Arg) in exon 53. In Le Guedard‐Mereuze et al. (2010), c.486‐14G>A was investigated in a splicing reporter minigene assay and was shown to result in creation of a new 3′AG acceptor splice site and as a result of this inclusion of intronic sequence in the open reading frame leading to a missense mutation (p.Met162Ile) and addition of 4 additional residues (between codon 162 and 163) and the authors speculate that the change will alter the structure of the *USH2A* protein. The ExAC database report that c.486‐14G>A is only observed in 2 of 120994 alleles consistent with the frequency of Usher syndrome.

The most frequent USH2 mutation was the c.2299delG mutation, which was identified in 56 alleles (45% of a total of 125 *USH2A* alleles with identified mutations). The mutation c.920_923dup was detected in five alleles (4%, 5/125). The mutations c.10561T>C, p.(Trp3521Arg) and c.1606T>C, p.(Cys536Arg) were identified in four alleles (3%, 4/125) each, and the four mutations c.2023C>T, p.(Gln675*), c.9120G>A, (p.(Trp3040*), c.11864G>A, p.(Trp3955*), and c.13316C>T, p.(Thr4439Ile) in three alleles each (2%). The rest of the mutations were identified only in one or two alleles each. All individuals were of Danish origin.

#### Mutation in a patient with USH3

In the combined Danish cohort, only one USH3 individual, the above described USH3‐1 (Table 1), was genotyped (homozygous for the mutation c.254‐1G>A in *CLRN1*). Her parents were consanguineous and of Syrian origin (Table 2; Table S3). The consequence of the c.254‐1G>A mutation is likely a skipping of exon 2, as it affects a splice site.

#### Molecular findings, in total

Full or partial molecular genetic diagnosis was obtained in 118 patients with USH, representing 100 probands and 18 affected family members. From the total cohort of 100 probands two or three mutations have so far been identified in 26 USH1, 58 USH2, and 1 USH3 individuals and one mutation has been identified in 6 individuals with USH1 and 9 individuals with USH2 (Tables S1–S3).

#### Clinical findings

Our entire cohort of 107 individuals (30 USH1, 76 USH2 and one USH3), were between 6 and 93 years of age (average age 49.9 years, median 51 years). All patients fulfilled the criteria for RP, nyctalopia, visual field constriction, retinal arteriolar attenuation, retinal pigmentation presenting as bone spicules, optic disc pallor, as well as severely reduced or flat standard full‐field ERG. The slit‐lamp examination showed cataract in 71% of the examined individuals; 18/30 of the persons with USH1 and 57/76 of the persons with USH2. The single USH3 individual did not have cataract at the age of 17. The 75 individuals with cataract were between 21 and 93 years of age with an average age of 55.7 years (median 54 years). The 32 individuals without cataract were between 6 and 93 years of age and the average age was 26.5 years (median 32 years).

Forty‐four individuals (14 USH1, 29 USH2 and one USH3) were examined by OCT, these individuals were between 6 and 93 years old with an average age of 38.8 (median 39.5). This examination revealed cystic macular edema in 52%: 8/14 USH1, 14/29 USH2, and in the single USH3 case (Tables S1–S3). The 23 individuals with macular edema were between 11 and 77 years old and the average age was 38 years (median 40 years). The 21 individuals with no macular edema were between 6 and 65 years of age (average 36 years; median 39 years). In our cohort there was no case with cystic macular edema led to macular atrophy or macular hole.

The olfactory function in all twelve examined individuals (2 USH1, 10 USH2) were within normal range in all. All had a score of 11 or 12 out of 12 possible.

## Discussion

The simultaneous investigation of all known USH genes by NGS technology is likely to facilitate the molecular diagnosis and broaden our view on the clinical features of the USH syndromes. However, even targeted NGS of all known USH genes does not lead to a molecular diagnosis in all cases. In subcohort 1b, two pathogenic variants were identified in 12 of 17 individuals (71%). Similar studies have achieved a similar percentage. Bonnet et al. (2011) identified two pathogenic variants in 41 of 54 USH patients (76%) and one mutation in 8 additional, in total they found mutations on 83% of the alleles (Bonnet et al. 2011). Le Quesne Stabej et al., identified two pathogenic variant in 112 of 172 USH patients (65%) and one in 27 USH patients (Le Quesne et al. 2012). In total they found mutations on 73% of the alleles. The reasons for absence of identification of two mutations in about 35–24% of the investigated patients in these studies may be either misdiagnosis, the presence of pathogenic variants outside the coding regions of the genes tested, or in genes not yet associated with USH. The recently identified USH genes, *CIB2*,* HARS* and *ABHD12* were not investigated, as they were not associated with USH, when the studies were initiated. However, mutations in these genes are probably very rare causes of USH (Jan 2013).

Furthermore, the clinical diagnosis of USH can be challenging. The various USH phenotypes may resemble other deaf‐blindness syndromes, the association between deafness and RP might be caused by unrelated genetic events, or the USH case may be atypical. Mutations in several genes may result in either USH or congenital deafness.

In most USH cases the differential diagnosis is easy mainly due to the degree of hearing impairment as shown by the audiogram. In a few patients, however, the clinical diagnosis may be ambiguous. In this study, the involved gene deviated in two patients. In cohort 1a, one person (USH3‐1), initially diagnosed with USH1, was found to be homozygous for the c.254‐1G>A mutation in the USH3 gene *CLRN1*. This patient did in fact present a phenotype, which differed from the typical USH3 phenotype, showing profound congenital hearing impairment and early onset of RP. However, she had progressive hearing impairment which is characteristic of USH3 and the underlying medical information from her early years was very sparse. Another person (USH2‐20), initially thought to have USH3, was found to have an *USH2A* genotype; c.[14384T>G(;)2299delG]. A reassessment of the clinical data made clear that USH2‐20 actually presented an unusual clinical picture with recognition of hearing impairment and RP after the age of 40. Thus, the specialized ophthalmological examinations should always be accompanied by molecular genetic examinations.

In the combined Danish USH cohort a total of 100 unrelated individuals have been genotyped. Two mutations were identified in 85 and only one mutation was found in the 15 remaining. As mutation screening was performed by screening of single genes or testing for known mutations (APEX) it is possible that the disease causing mutations in these 15 patients are present in other USH genes not screened, and the identified mutations only are coincidental findings. In the Danish USH1 cohort mutations in *CDH23* were mainly identified in individuals of non‐Danish origin, whereas mutations in *MYO7A,* mostly were identified in individuals with Danish origin. Compared to the mutation pattern obtained in USH1 cohorts from other countries, *MYO7A* mutations accounted for a larger fraction of the mutations in the Danish cohort. In cohorts from Britain, France and Italy, 61–63.3% of the genotyped individuals with USH1 had mutations in *MYO7A* (Roux et al. 2011; Le Quesne et al. 2012). The mutation, c.93C>A (p.Cys31*) in *MYO7A,* accounts for 33% of all USH1 alleles with identified mutations in Denmark. Mutations were identified in more than 100 individuals with USH1 from France, but the p.Cys31* mutation was not identified in any of these individuals (Roux et al. 2006, 2011; Bonnet et al. 2011). Le Quesne et al. (2012) identified a single family with p.Cys31* mutation in a cohort of 47 individuals with USH1 from Britain. The high allele frequency of this mutation among Danish individuals with USH1 suggests that the p.Cys31* mutation is a Danish founder mutation, in agreement with previous observations (Janecke et al. 1999). The presence of the p.Cys31* mutation might also explain the higher fraction of mutations in *MYO7A* in Denmark compared with other countries.

The fraction of individuals with mutations in *CDH23* and *USH1C* varied in different countries, from 13–20% and 4.5–16%, respectively (Roux et al. 2011; Le Quesne et al. 2012).

In the Danish cohort of individuals with USH2, mutations were solely identified in *USH2A*, in agreement with previous studies from several other countries, showing that *USH2A* is the main contributor and *DFNB31* and *ADGRV1* represents minor contributors (Garcia‐Garcia et al. 2011; Le Quesne et al. 2012). In the entire cohort of 100 individuals, mutations in *CLRN1* were identified only in one individual of non‐Danish origin. Similarly, *CLRN1* mutations are also rare in most other populations (Bonnet et al. 2011; Le Quesne et al. 2012). The USH3 phenotype and especially molecularly verified cases are rare, as many patients with a clinical diagnosis of USH3 in fact turn out to have mutations in USH2 (Bonnet et al. 2011; Le Quesne et al. 2012). Only in Finland and among Ashkenazi Jews, the USH3 phenotype with mutations in *CLRN1* is frequent and accounts for 40% of all USH cases (Joensuu et al. 2001; Ness et al. 2003; Vastinsalo et al. 2006; Herrera et al. 2008).

The mutation c.2299delG (p.Glu767Serfs*21) in *USH2A* had an allele frequency of 45% in the Danish USH2 cohort. Other studies also report that this mutation is frequent, but the allele frequency varies. Le Quesne et al. (2012) reported a c.2299delG allele frequency of 33.7% in a cohort of 96 British USH2 individuals with identified mutations. In Spain, the allele frequency of c.2299delG was only 15% (Garcia‐Garcia et al. 2011). The allele frequency thus varies from being high in Northern Europe (45%) to being low in Southern Europe (15%). The higher frequency in Northern Europe could indicate that the mutation arose many generations back in a common ancestor from Scandinavia and from here spread to Southern Europe via migration. Alternatively, as proposed by Dreyer et al. (2001), “it is tempting to speculate that the c.2299delG mutation results from an old mutational event that happened to arise on the most common haplotype in the present European genetic background and that was spread, though migration and subsequent founder effects, throughout Europe”.

**Figure 2:**
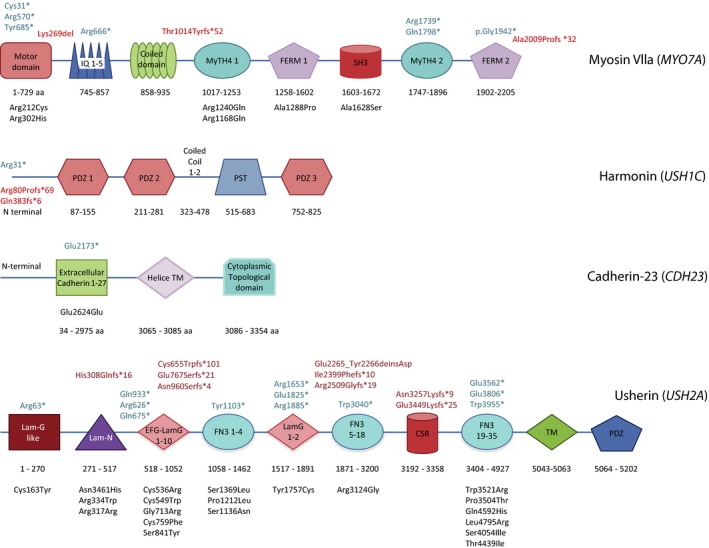
Predicted structures of the affected proteins. Location of affected residues in 100 Danish USH individuals, are indicated. Myosin VIIa contains eight functional domains; the motor domain, the IQ motif containing five leucine‐glutamine repeats, the coiled–coil (CCl) domain, two MyTH4 repeats separated by the src homology‐3 (SH3) domain and a FERM domain. The harmonin transcripts can be divided into three isoforms, which differ in the composition of the C‐terminal part of the protein. The largest isoform (b) contains three PDZ domains, two coiled–coil domains and a PST domain, the shortest isoform (c) contains two PDZ domains and one coiled–coil domain. Cadherin‐23 contains three domains, an extracellular cadherin 1–27 domain, a helical transmembrane domain and a cytoplasmic domain. Usherin contains ten different domains: one laminin G‐like domain, one laminin N‐terminal domain, ten laminin‐type EGF‐like domains, 35 fibronectin type III domains (FN3), one transmembrane domain (TM), one PDZ domain and one cysteine‐rich domain. Mutations in blue are nonsense mutations, mutations in black are missense mutations and mutations in red are frameshift mutations.

The location of the affected residues in the predicted structure of the proteins, are shown in Figure 2. In *MYO7A*, affected residues were located in all domains except in the coiled–coil domain. The motor domain was the most frequently affected as also reported in other studies (Petit 2001). This domain regulates ATP hydrolysis and interacts with actin filaments (Sata et al. 1997). Previous studies have suggested that it affects protein stability (Hasson et al. 1997) and motor function (Watanabe et al. 2008). In *USH1C*, only mutations affecting the N‐terminus, leading to truncation of the protein, were identified. In *CDH23*, we only identified mutations affecting residues located in the extracellular cadherin 1–2 domain, which plays a major role in the function of the protein and which also interacts with *PCDH15*. This interaction is critical for sound detection via mechano‐transduction by the sensory hair cells. The majority of the *USH2A* mutations affect residues located in the laminin EGF‐like domains and in the FN3 domains. Laminin EGF‐like domains are thought to play a role in the folding of the protein, as mutations in this module result in abnormal *USH2A* proteins (Dreyer et al. 2000). FN3 domains are found in extracellular proteins, and play a role in the spatial arrangement of other domains and in the formation of protein–protein interfaces.

Seventy‐one percent of the examined patients had cataract and 52% had macular edema. This underscores the importance of longitudinal ophthalmological monitoring for possible therapeutic action to preserve central vision as long as possible.

We only had opportunity to examine the olfactory function in a very few individuals, mainly individuals with USH2. This is partly due to the difficult communication, which requires the assistance of personnel skilled in nonverbal communication. The results of our examination should therefore only be considered as a pilot test. Data regarding the association of USH with olfactory dysfunction are conflicting. Zrada et al., identified olfactory dysfunction in 11 out of 22 individuals with USH (5 USH1 and 6 USH2) by the University of Pennesylvania Smell Identification Test (UPSIT; Zrada et al. 1996), while Seeliger et al., could not identify any olfactory dysfunction in their cohort of 39 persons with USH (8 USH1, 31 USH2; Seeliger et al. 1999). However, the majority of USH proteins are expressed in olfactory cells (Wolfrum et al. 1998). Recently the USH proteins encoded by *MYO7A, USH1C; USH1D, USH1F, USH1G, USH2A, USH2C* and *USH2D* were shown to be expressed in the murine olfactory epithelium (OE). Furthermore, investigation of five murine models with mutations in *MYO7A, USH1C, CDH23, PCDH15* and *USH1G*, respectively, showed that mutations in *USH1C* and *USH1G* lead to olfactory impairment in the mouse (Jansen et al. 2016). Thus, it is not unlikely that olfactory function might be impaired in some USH individuals.

Targeted NGS of USH combined with analysis for large genomic rearrangements has improved molecular genetic diagnosis in USH. An early genetic diagnosis is essential for the patient regarding the possibilities of cochlear implant, genetic counseling, and future advanced treatments.

## Conflict of Interest

The authors declare no conflict of interests.

## Supporting information


**Table S1.** Mutations and clinical information of Danish individuals with USH1. NI: not identified; **NA:** Information not available; **−:** absent, **+:** present; **HI:** hearing impairment; **Seq:** sequencing**, APEX: arrayed primer extension microarray; mutations in bold:** novel according to HGMDprof and LOVD USH database 161015; Accession numbers: *MYO7A* (NM_000260.3); *CDH23* (NM_022124.5); *USH1C* (NM_005709.3). Gray‐marked individuals indicate a family member. Information about onset of night blindness is in several cases based on information obtained from the patient. It was often difficult for the patient to set the exact age at onset, and may thus be associated with inaccuracy.

**Table S2.** Mutations and clinical information of Danish individuals with USH2. All mutations are identified in *USH2A* and in patients with Danish origin. **NI:** not identified; **NA:** information not available; **−:** absent, **+:** present; **HI:** hearing impairment; **Seq:** sequencing, **APEX: arrayed primer extension microarray; mutations in bold:** novel according to HGMDprof and LOVD USH database 161015; Accession number: *USH2A* (NM_206933.2). Gray‐marked individuals indicate a family member. Information about onset of night blindness is en several cases based on information obtained from the patient. In several cases it was difficult for the patient to set the exact age at onset, and may thus be associated with inaccuracy.

**Table S3.** Clinical data and mutations identified in an individual with USH3. −: absent, +: present; mutations in bold: novel according to HGMDprof and LOVD USH database 161015; Accession number: *CLRN1* (NM_174878.2).

**Table S4**. Primer list and PCR program. Table includes all primers used for verification of the variants identified by NGS and primers for Sanger sequencing of the intronic variant *c.7595‐2144A>G* in *USH2A*.
