# Supplementary material for: Usher syndrome in Denmark: mutation spectrum and some clinical observations
Source: Mol Genet Genomic Med. 2016 Jun 28;4(5):527–39. doi: 10.1002/mgg3.228 (PMC5023938; doi:10.1002/mgg3.228)
Supplement: Supplementary file 1 — Table S1. Mutations and clinical information of Danish individuals with USH1. NI: not identified; NA: Information not available; −: absent, +: present; HI: hearing impairment; Seq: sequencing, APEX: arrayed primer extension microarray; mutations in bold: novel according to HGMDprof and LOVD USH database 161015; Accession numbers: MYO7A (NM_000260.3); CDH23 (NM_022124.5); USH1C (NM_005709.3). Gray‐marked individuals indicate a family member. Information about onset of night blindness is in several cases based on information obtained from the patient. It was often difficult for the patient to set the exact age at onset, and may thus be associated with inaccuracy. [file MGG3-4-527-s001.docx]

| **Patient** | **Gender** | **Onset of night blindness** | **Macula edema** | **Cataract** | **Audiologi: Congenital severe to profound HI (USH1)** | **Gene** | **Allele 1** | **Predicted protein** | **Exon** | **Allele 2** | **Predicted protein** | **Exon** | **Method** | **Origin** | **Consanguinity** | **Remarks** | **Patient published** |
| --- | --- | --- | --- | --- | --- | --- | --- | --- | --- | --- | --- | --- | --- | --- | --- | --- | --- |
| USH1-1 | F | NA | NA | NA | NA | *MYO7A* | c.805_807del | p.Lys269del | 8 | c.93C>A | p.Cys31* | 3 | Targeted NGS of USH genes (Ot5334) | Denmark | - | - | Present study |
| USH1-2 | F | 10 yrs | - | - | NA | *MYO7A* | c.93C>A | p.Cys31* | 3 | c.93C>A | p.Cys31* | 3 | Targeted NGS of USH genes (Ot9096) | Denmark | - | Vestibular dysfunction | Present study |
| USH1-3 | F | 39 yrs | NA | + | NA | *MYO7A* | c.3503G>A | p.Arg1168Gln | 27A | c.93C>A | p.Cys31* | 3 | Targeted NGS of USH genes (Ot5342) | Denmark | - | - | Present study |
| USH1-4 | F | 37 yrs | - | NA | NA | *MYO7A* | c.4882G>T | p.Ala1628Ser | 36 | c.4882G>T | p.Ala1628Ser | 36 | APEX | Denmark | - | - | Tranebjaerg et al 2011 |
| USH1-5 | F | 12 yrs | NA | + | NA | *MYO7A* | c.93C>A | p.Cys31* | 3 | c.93C>A | p.Cys31* | 3 | APEX /*MYO7A* seq | Denmark | - | - | Tranebjaerg et al 2011 |
| USH1-5A | F | NA | NA | NA | NA | *MYO7A* | c.93C>A | p.Cys31* | 3 | c.93C>A | p.Cys31* | 3 | APEX / *MYO7A* seq | Denmark | - | - | Tranebjaerg et al 2011 |
| USH1-6 | M | NA | - | + | NA | *CDH23* | **c.6517G>T** | **p.Glu2173*** | 48 | **c.6517G>T** | **p.Glu2173*** | 48 | Targeted NGS of USH genes (Ot9087) | Denmark | + | - | Present study |
| USH1-6B | M | 3-4 yrs | NA | + | NA | *CDH23* | **c.6517G>T** | **p.Glu2173*** | 48 | **c.6517G>T** | **p.Glu2173*** | 48 | Targeted NGS of USH genes (Ot200-489) | Denmark | - | - | Present study |
| USH1-7 | M | Always | NA | - | + | *MYO7A* | c.93C>A | p.Cys31* | 3 | c.3719G>A | p.Arg1240Gln | 29 | *MYO7A* seq | Denmark | - | Vestibular dysfunction | Tranebjaerg et al 2011, Janecke et al 1999 |
| USH1-8 | M | NA | NA | - | NA | *CDH23* | c.4489-2A>C (IVS35-2A>C) | splice defect | intron 35 | c.4360-2A>C (IVS35-2A>C) | splice defect | intron 35 | APEX / *MYO7A* seq | Irak | + | - | Tranebjaerg et al 2011 |
| USH1-9 | M | 41 yrs | NA | + | + | *MYO7A* | c.93C>A | p.Cys31* | 3 | NI | NI |  | *MYO7A* seq | Denmark | - | Vestibular dysfunction. | Janecke et al 1999 |
| USH1-10 | M | NA | NA | + | + | *MYO7A* | c.1555-8C>G (IVS13-8C>G) | splice defect | intron 13 | NI | NI |  | *MYO7A* seq | Denmark | - | - | Cremers et al 2007 |
| USH1-11 | F | 11 yrs | NA | + | + | *MYO7A* | c.5215C>T | p.Arg1739* | 38 | c.5824G>T | p.Gly1942* | 42 | APEX/ *MYO7A* seq | Denmark | - | - | Tranebjaerg et al 2011 |
| USH1-12 | F | NA | + | + | NA | *MYO7A* | c.93C>A | p.Cys31* | 3 | NI | NI |  | *MYO7A* seq | Denmark | - | - | Janecke et al 1999 |
| USH1-13 | F | 8 yrs | + | - | NA | *USH1C* | **c.1146dupA** | **p.Gln383fs*6** | 14 | **c.1146dupA** | **p.Gln383fs*6** | 14 | *USH1C* seq | Denmark | - | Vestibular dysfunction | Present study |
| USH1-14 | F | 5 yrs | - | NA | NA | *MYO7A* | c.3040_3043delins TACTTCCAGGGGACA | p.Thr1014Tyrfs*52 | 24 | c.3040_3043delins TACTTCCAGGGGACA | p.Thr1014Tyrfs*52 | 24 | *MYO7A* seq | Turkey | - | Vestibular dysfunction | Tranebjaerg et al 2011 |
| USH1-15 | M | 5-6 yrs | NA | + | NA | *MYO7A* | c.4882G>T | p.Ala1628Ser | 36 | NI | NI |  | *MYO7A* seq | Denmark | - | Vestibular dysfunction | Janecke et al 1999 |
| USH1-16 | M | 8 yrs | NA | + | NA | *MYO7A* | c.93C>A | p.Cys31* | 3 | c.93C>A | p.Cys31* | 3 | *MYO7A* seq | Denmark | - | - | Janecke et al 1999 |
| USH1-17 | M | NA | NA | + | NA | *USH1C* | c.91C>A | p.Arg31* | 2 | c.238dup | p.Arg80Profs*69 | 3 | APEX | Denmark | - | - | Cremers et al 2007 |
| USH1-18 | M | 10 yrs | - | - | + | *MYO7A* | c.93C>A | p.Cys31* | 3 | c.5392C>T | p.Gln1798* | 39 | APEX | Denmark | - | - | Cremers et al 2007 |
| USH1-19 | M | 36 yrs | NA | + | NA | *USH1C* | c.238dup | p.Arg80Profs*69 | 3 | c.238dup | p.Arg80Profs*69 | 3 | APEX | Denmark | - | - | Janecke et al 1999 |
| USH1-20 | F | 14 yrs | + | - | NA | *CDH23* | c.6050-9G>A (IVS45-9G>A) | splice defect | intron 46 | c.6050-9G>A (IVS45-9G>A) | splice defect | intron 46 | *CDH23* seq | Pakistan | + | - | Tranebjaerg et al 2011 |
| USH1-21 | M | NA | - | - | + | *CDH23* | c.7872G>A | p.Glu2624Glu | 56 | c.7872G>A | p.Glu2624Glu | 56 | APEX | Pakistan | + | - | Tranebjaerg et al 2011 |
| USH1-21 A | F | 10 yrs | + | - | + | *CDH23* | c.7872G>A | p.Glu2624Glu (splice defect) | 56 | c.7872G>A | p.Glu2624Glu | 56 | APEX | Pakistan | + | - | Tranebjaerg et al 2011 |
| USH1-22 | M | 1 yrs | NA | - | NA | *MYO7A* | c.93C>A | p.Cys31* | 3 | c.93C>A | p.Cys31* | 3 | *MYO7A* seq | Denmark | - | Vestibular dysfunction | Janecke et al 1999 |
| USH1-23 | F | 17 yrs | + | - | + | *MYO7A* | c.634C>T | p.Arg212Cys | 7 | c.634C>T | p.Arg212Cys | 7 | APEX | Denmark | - | - | Cremers et al 2007 |
| USH1-24 | F | 10-12 yrs | NA | + | NA | *MYO7A* | c.93C>A | p.Cys31* | 3 | c.93C>A | p.Cys31* | 3 | *MYO7A* seq | Denmark | - | - | Janecke et al 1999 |
| USH1-25 | F | 21 yrs | + | + | + | *MYO7A* | c.905G>A | p.Arg302His | 9 | NI | NI |  | APEX | Denmark | - | - | Cremers et al 2007 |
| USH1-26 | M | 8 yrs | NA | + | + | *MYO7A* | c.1996C>T | p.Arg666* | 17 | c.3862G>C | p.Ala1288Pro | 30 | *MYO7A* seq | Denmark | - | - | Janecke et al 1999 |
| USH1-27 | M | 43 yrs | NA | + | + | *MYO7A* | c.3719G>A | p.Arg1240Gln | 29 | NI | NI |  | APEX | Denmark | - | - | Cremers et al 2007 |
| USH1-28 | F | 8 yrs | NA | + | + | *MYO7A* | c.93C>A | p.Cys31* | 3 | c.6025delG | p.Ala2009Profs*32 | 44 | APEX | Denmark | - | - | Cremers et al 2007 |
| USH1-29 | F | NA | + | - | + | *MYO7A* | c.2055C>A | p.Tyr685* | 17 | c.3719G>A | p.Arg1240Gln | 29 | APEX/ *MYO7A* seq | Denmark | - | Vestibular dysfunction | Janecke et al 1999 |
| USH1-29A | M | 4 yrs | + | - | + | *MYO7A* | c.2055C>A | p.Tyr685* | 17 | c.3719G>A | p.Arg1240Gln | 29 | APEX/ *MYO7A* seq | Denmark | - | Vestibular dysfunction |  |
| USH1-30 | F | NA | NA | NA | + | *MYO7A* | c.1708C>T | p.Arg570* | 15 | c.1708C>T | p.Arg570* | 15 | Targeted NGS of USH genes (Ot5849) | Sri Lanka | - | - | Present study |
| USH1-31 | F | NA | NA | + | NA | *MYO7A* | c.93C>A | p.Cys31* | 3 | c.93C>A | p.Cys31* | 3 | Targeted NGS of USH genes (Ot5855) | Denmark | - | - | Present study |
| USH1-35 | F | NA | - | - | NA |  | NI |  | NI |  |  |  | Targeted NGS of USH genes (Otogenetics) Ot5343 | Turkey | - | - | Present study |
| USH1-36 | F | NA | - | + | + | *CDH23* | **c.3862C>T** | **p.Gln1288*** | 32a | **c.3862C>T** | **p.Gln1288*** | 32a | Targeted NGS of USH genes (Otogenetics) Ot9095 | Sri Lanka | - | - | Present study |
| USH1-37 | F | NA | - | - | + |  | NI |  | NI |  |  |  | Targeted NGS of USH genes (Otogenetics) Ot5341 | Afghanistan | - | - | Present study |
| USH1-38 | F | NA | NA | NA | NA |  | NI |  | NI |  |  |  | Targeted NGS of USH genes (Otogenetics) Ot5344 | Denmark | - | - | Present study |
