# Supplementary material for: Usher syndrome in Denmark: mutation spectrum and some clinical observations
Source: Mol Genet Genomic Med. 2016 Jun 28;4(5):527–39. doi: 10.1002/mgg3.228 (PMC5023938; doi:10.1002/mgg3.228)
Supplement: Supplementary file 2 — Table S2. Mutations and clinical information of Danish individuals with USH2. All mutations are identified in USH2A and in patients with Danish origin. NI: not identified; NA: information not available; −: absent, +: present; HI: hearing impairment; Seq: sequencing, APEX: arrayed primer extension microarray; mutations in bold: novel according to HGMDprof and LOVD USH database 161015; Accession number: USH2A (NM_206933.2). Gray‐marked individuals indicate a family member. Information about onset of night blindness is en several cases based on information obtained from the patient. In several cases it was difficult for the patient to set the exact age at onset, and may thus be associated with inaccuracy. [file MGG3-4-527-s002.docx]

| **Patient** | **Gender** | **Onset of night blindness** | **Macular edema** | **Cataract** | **Audiology : Congenital moderate to severe HI (USH2)** | **Allele 1** | **Predicted protein** | **Exon** | **Allele 2** | **Predicted protein** | **Exon** | **Method** | **Consanguinity** | **Remarks** | **Patient published** |
| --- | --- | --- | --- | --- | --- | --- | --- | --- | --- | --- | --- | --- | --- | --- | --- |
| USH2-1 | F | 30 yrs | + | - | + | c.1606T>C | p.Cys536Arg | 9 | c.1606T>C | p.Cys536Arg | 9 | Targeted NGS of USH genes (Ot9089) | - | - | Present study |
| USH2-2 | M | 60 yrs | + | - | NA | c.9370A>G | p.Arg3124Gly | 47 | c.2299delG | p.Glu767Serfs*21 | 13 | Targeted NGS of USH genes (Ot9086) | - | - | Present study |
| USH2-3 | M | 15 yrs | + | + | NA | c.2299delG | p.Glu767Serfs*21 | 13 | c.2299delG | p.Glu767Serfs*21 | 13 | Targeted NGS of USH genes (Ot9091) | - | - | Present study |
| USH2-4 | M | 16 yrs | NA | + | + | c.9770dup | p.Asn3257Lysfs*9 | 50 | c.2299delG | p.Glu767Serfs*21 | 13 | *USH2A* seq | - | - | Dreyer et al 2000 Dreyer et al 2008. |
| USH2-4a (D58) | F | 4 yrs | NA | + | + | c.9770dup | p.Asn3257Lysfs*9 | 50 | c.2299delG | p.Glu767Serfs*21 | 13 | *USH2A* seq | - | - | Dreyer et al 2000, Dreyer et al 2008 |
| USH2-5 (D61) | F | Always | + | NA | + | c.3309C>A | p.Tyr1103* | 16 | MLPA:c.4628_4987del | p.Gly1543_Pro1662del | 22-24 | *USH2A* seq | - | - | Present study, Dad et al 2015 |
| USH2-6 (D73) | M | 21 yrs | NA | - | NA | c.2299delG | p.Glu767Serfs*21 | 13 | NI | NI |  | *USH2A* seq | Possibly | - | Dreyer et al 2000 Dreyer et al 2008. |
| USH2-6a(D71) | M | NA | NA | NA | NA | c.2299delG | p.Glu767Serfs*21 | 13 | c.2276G>T | p.Cys759Phe | 13 | *USH2A* seq | - | - | Dreyer et al 2000, Dreyer et al 2008 |
| USH2-7 | M | Childhood | - | - | NA | c.10684G>T | p.Glu3562* | 54 | MLPA:c.672_1840del | p.Ser224Argfs*5 | 4-10 | Targeted NGS (Ot5345) | - | - | Present study, Dad et al 2015 |
| USH2-8 (D69) | F | 19 yrs | - | - | NA | c.2299delG | p.Glu767Serfs*21 | 13 | MLPA:c.672_1840del | p.Ser224Argfs*5 | 4-10 | *USH2A* seq | - | - | Dreyer et al 2000,  Dreyer et al 2008, Dad et al 2015 |
| USH2-9 | M | 11-12 yrs | + | - | + | c.10561T>C | p.Trp3521Arg | 53 | c.486-14G>A | p? | intron 2 | Targeted NGS (Ot9094 ) | - | - | Present study |
| USH2-10 | F | 13 yrs | NA | - | NA | c.10510C>A | p.Pro3504Thr | 53 | c.2299delG | p.Glu767Serfs*21 | 13 | *USH2A* seq | - | - | Dreyer et al 2000, Dreyer et al 2008 |
| USH2-10a (D08) | F | NA | NA | NA | + | c.10510C>A | p.Pro3504Thr | 53 | c.2299delG | p.Glu767Serfs*21 | 13 | *USH2A* seq | - | - | Dreyer et al 2000, Dreyer et al 2008 |
| USH2-11 | M | 10-12 yrs | - | - | NA | c.2299delG | p.Glu767Serfs*21 | 13 | c.4106C>T | p.Ser1369Leu | 19 | APEX | - | - | Present study |
| USH2-12 (D55) | F | NA | NA | + | NA | c.13776G>C | p.Gln4592His | 63 | c.11864G>A | p.Trp3955* | 61 | *USH2A* seq | - | - | Dreyer et al 2000, Dreyer et al 2008 |
| USH2-12a (D36) | M | NA | NA | + | NA | c.13776G>C | p.Gln4592His | 63 | c.11864G>A | p.Trp3955* | 61 | *USH2A* seq | - | - | Dreyer et al 2000, Dreyer et al 2008 |
| USH2-13 (D21) | F | 31 yrs | + | + | + | c.2023C>T; c.2797C>T | p.Gln675*; p.Gln933* | 12 | c.488G>A | p.Cys163Tyr | 12 | *USH2A* seq | - | - | Dreyer et al 2000, Dreyer et al 2008, Tranebjaerg et al 2011 |
| USH2-13a | M | Childhood | - | + | NA | c.2023C>T; c.2797C>T | p.Gln675*; p.Gln933* | 12 | c.488G>A | p.Cys163Tyr | 12 | *USH2A* seq | - | - | Dreyer et al 2000, Dreyer et al 2008, Tranebjaerg et al 2011 |
| USH2-14 (D54) | F | NA | NA | + | NA | c.2299delG | p.Glu767Serfs*21 | 13 | c.3635C>T | p.Pro1212Leu | 17 | *USH2A* seq | - | - | Dreyer et al 2000, Dreyer et al 2008, Tranebjaerg et al 2011 |
| USH2-15 (D-19) | M | 15 yrs | NA | + | + | c.2299delG | p.Glu767Serfs*21 | 13 | c.2299delG | p.Glu767Serfs*21 | 13 | *USH2A* seq | - | - | Dreyer et al 2000, Dreyer et al 2008 |
| USH2-15a (D37) | F | 26 yrs | NA | - | NA | c.2299delG | p.Glu767Serfs*21 | 13 | c.2299delG | p.Glu767Serfs*21 | 13 | *USH2A* seq | - | - | Dreyer et al 2000, Dreyer et al 2008 |
| USH2-16 (D68) | F | Childhood | - | + | Yes | c.920_923dup | p.His308Glnfs*16 | 6 | c.920_923dup | p.His308Glnfs*16 | 6 | *USH2A* seq | - | - | Dreyer et al 2000, Dreyer et al 2008, |
| USH2-16a | M | 30 yrs | - | + | NA | c.920_923dup | p.His308Glnfs*16 | 6 | c.920_923dup | p.His308Glnfs*16 | 6 | *USH2A* seq | - | - | Dreyer et al 2000, Dreyer et al 2008 |
| USH2-17 | M | 52 yrs | - | + | NA | c.2299delG | p.Glu767Serfs*21 | 13 | c.2299delG | p.Glu767Serfs*21 | 13 | APEX | - | - | Present study |
| USH2-18 | F | 12 yrs | + | - | NA | c.2299delG | p.Glu767Serfs*21 | 13 | c.13316C>T | p.Thr4439Ile | 63 | APEX/*USH2A* seq | - | - | Tranebjaerg et al 2011 |
| USH2-19 (D40) | M | Childhood | NA | + | + | c.2299delG | p.Glu767Serfs*21 | 13 | c.2299delG | p.Glu767Serfs*21 | 13 | *USH2A* seq | - | Vestibular dysfunction | Dreyer et al 2000, Dreyer et al 2008 |
| USH2-20 | F | 35 yrs | - | >40 yrs | + | c.14384T>G | p.Leu4795Arg | 66 | c.2299delG | p.Glu767Serfs*21 | 13 | Targeted NGS of USH genes (Ot9092) | - | Vestibular dysfunction | Present study |
| USH2-21 | M | 16-17 yrs | + | + | + | c.2299delG | p.Glu767Serfs*21 | 13 | NI | NI |  | *USH2A* seq | - | - | Present study |
| USH2-22 (D26) | F | 8-9 yrs | - | + | + | c.11416G>T | p.Glu3806* | 59 | c.13316C>T | p.Thr4439Ile | 63 | *USH2A* seq | - | - | Dreyer et al 2000, Dreyer et al 2008 |
| USH2-23 (D33) | M | 33 yrs | NA | + | NA | c.2299delG | p.Glu767Serfs*21 | 13 | c.1606T>C | p.Cys536Arg | 9 | *USH2A* seq | - | - | Dreyer et al 2000, Dreyer et al 2008 |
| USH2-23a (D50) | F | 37 yrs | + | + | + | c.2299delG | p.Glu767Serfs*21 | 13 | c.1606T>C | p.Cys536Arg | 9 | *USH2A* seq | - | - | Tranebjaerg et al 2011 |
| USH2-23b (D50) | M | 31 yrs | NA | + | + | c.2299delG | p.Glu767Serfs*21 | 13 | c.1606T>C | p.Cys536Arg | 9 | *USH2A* seq | - | - | Tranebjaerg et al 2011 |
| USH2-24 (D53) | M | 15 yrs | NA | + | + | c.12161G>T | p.Ser4054Ile | 62 | c.6795_6797delATA | p.Glu2265_Tyr2266 delinsAsp | 35 | *USH2A* seq | - | - | Dreyer et al 2000, Dreyer et al 2008 |
| USH2-25 (D60) | F | 30 yrs | NA | + | + | c.9120G>A | p.Trp3040* | 46 | NI | NI |  | *USH2A* seq | - | - | Dreyer et al 2000, Dreyer et al 2008 |
| USH2-26 (D20) | F | 20 yrs | NA | + | + | c.1036A>C | p.Asn346His | 6 | NI | NI |  | *USH2A* seq | - | - | Dreyer et al 2000, Dreyer et al 2008 |
| USH2-27 (D44) | M | NA | + | + | + | c.2299delG | p.Glu767Serfs*21 | 13 | c.2299delG | p.Glu767Serfs*21 | 13 | *USH2A* seq | - | - | Dreyer et al 2000, Dreyer et al 2008 |
| USH2-28 | M | 28 yrs | NA | + | + | c.2299delG | p.Glu767Serfs*21 | 13 | c.2299delG | p.Glu767Serfs*21 | 13 | *USH2A* seq | - | - | Dreyer et al 2000, Dreyer et al 2008 |
| USH2-28a (D02) | M | NA | NA | + | NA | c.2299delG | p.Glu767Serfs*21 | 13 | c.2299delG | p.Glu767Serfs*21 | 13 | *USH2A* seq | - | - | Dreyer et al 2000, Dreyer et al 2008 |
| USH2-29 | M | 27 | + | + | + | c.2522C>A | p.Ser841Tyr | 13 | NI | NI |  | APEX | - | - | Present study |
| USH2-30 (D48) | M | 25 yrs | NA | + | + | c.2299delG | p.Glu767Serfs*21 | 13 | c.2299delG | p.Glu767Serfs*21 | 13 | *USH2A* seq | - | - | Dreyer et al 2000, Dreyer et al 2008 |
| USH2-31 (D32) | M | 15 yrs | - | + | + | c.2299delG | p.Glu767Serfs*21 | 13 | c.12161G>T | p.Ser4054Ile | 62 | *USH2A* seq | - | - | Dreyer et al 2000, Dreyer et al 2008 |
| USH2-32 (D25) | M | 24 yrs | NA | + | + | c.2299delG | p.Glu767Serfs*21 | 13 | c.1000C>T | p.Arg334Trp | 6 | *USH2A* seq | - | - | Dreyer et al 2000, Dreyer et al 2008 |
| USH2-33(D52) | M | NA | - | + | + | c.2299delG | p.Glu767Serfs*21 | 13 | c.2299delG | p.Glu767Serfs*21 | 13 | *USH2A* seq | - | - | Dreyer et al 2000, Dreyer et al 2008 |
| USH2-34 (D63) | F | 13 yrs | NA | - | + | **c.1647T>G** | **p.Cys549Trp** | 10 | c.7524delT | p.Arg2509Glyfs*19 | 40 | *USH2A* seq | - | - | Present study |
| USH2-35 | F | 10 yrs | - | - | + | c.2299delG | p.Glu767Serfs*21 | 13 | c.2299delG | p.Glu767Serfs*21 | 13 | APEX | - | - | Tranebjaerg et al 2011 |
| USH2-36 (D07) | F | 20 yrs | - | + | + | c.2299delG | p.Glu767Serfs*21 | 13 | c.2797C>T | p.Gln933* | 13 | *USH2A* seq | - | - | Dreyer et al 2000, Dreyer et al 2008 |
| USH2-37 (D03) | M | NA | NA | + | + | c.2878_2879delAA | p.Asn960Serfs*4 | 14 | c.2878_2879delAA | p.Asn960Serfs*4 | 14 | *USH2A* seq | - | - | Dreyer et al 2000, Dreyer et al 2008 |
| USH2-38 | F | 16 yrs | + | + | + | c.949C>A | p.Arg317Arg | 6 | c.2028C>A | p.Cys676* | 12 | *USH2A* seq | - | - | Tranebjaerg et al 2011 |
| USH2-39 (D39) | F | 21 yrs | NA | + | + | c.2299delG | p.Glu767Serfs*21 | 13 | c.10561T>C | p.Trp3521Arg | 53 | *USH2A* seq | - | - | Dreyer et al 2000, Dreyer et al 2008 |
| USH2-40 (D38) | M | 19 yrs | NA | - | NA | c.11864G>A | p.Trp3955* | 61 | c.10561T>C | p.Trp3521Arg | 53 | *USH2A* seq | - | - | Dreyer et al 2000, Dreyer et al 2008 |
| USH2-41 (D17) | F | 30 yrs | NA | + | NA | c.2299delG | p.Glu767Serfs*21 | 13 | c.2137G>C | p.Gly713Arg | 12 | *USH2A* seq | - | - | Dreyer et al 2000, Dreyer et al 2008 |
| USH2-42 (D05) | F | NA | NA | + | NA | c.2299delG | p.Glu767Serfs*21 | 13 | c.9120G>A | p.Trp3040* | 46 | *USH2A* seq | - | - | Dreyer et al 2000, Dreyer et al 2008 |
| USH2-43 (D10) | M | 13-14 yrs | NA | + | + | c.9120G>A | p.Trp3040* | 13 | c.187C>T | p.Arg63* | 2 | *USH2A* seq | - | - | Dreyer et al 2000, Dreyer et al 2008 |
| USH2-43a (D06) | M | 20 yrs | NA | + | + | c.1920G>A | p.Trp3040* | 13 | c.187C>T | p.Arg63* | 2 | *USH2A* seq | - | - | Dreyer et al 2000, Dreyer et al 2008 |
| USH2-44 (D18) | F | Childhood | NA | + | + | c.2299delG | p.Glu767Serfs*21 | 13 | c.2299delG | p.Glu767Serfs*21 | 13 | *USH2A* seq | - | - | Dreyer et al 2000, Dreyer et al 2008 |
| USH2-45 (D42) | F | NA | NA | + | NA | c.1876C>T | p.Arg626* | 11 | c.4957C>T | p.Arg1653* | 24 | *USH2A* seq | - | - | Dreyer et al 2000, Dreyer et al 2008 |
| USH2-46 (D24) | F | 6 yrs | NA | + | NA | c.2299delG | p.Glu767Serfs*21 | 13 | c.10561T>C | p.Trp3521Arg | 53 | *USH2A* seq | - | - | Dreyer et al 2000, Dreyer et al 2008 |
| USH2-47 (D22) | M | 38 yrs | NA | + | + | c.2299delG | p.Glu767Serfs*21 | 13 | c.1606T>C | p.Cys536Arg | 9 | *USH2A* seq | - | - | Dreyer et al 2000, Dreyer et al 2008 |
| USH2-47a (D16) | F | Always | NA | + | + | c.2299delG | p.Glu767Serfs*21 | 13 | c.1606T>C | p.Cys536Arg | 9 | *USH2A* seq | - | - | Dreyer et al 2000, Dreyer et al 2008 |
| USH2-48 (D65) | F | NA | NA | - | + | c.13316C>T | p.Thr4439Ile | 63 | NI | NI |  | *USH2A* seq | - | - | Dreyer et al 2000, Dreyer et al 2008 |
| USH2-48a (D64) | F | NA | NA | - | + | c.13316C>T | p.Thr4439Ile | 63 | NI | NI |  | *USH2A* seq | - | - | Dreyer et al 2000, Dreyer et al 2008 |
| USH2-49 (D14) | M | 17-18 yrs | NA | + | + | c.920_923dup | p.His308Glnfs*16 | 6 | c.920_923dup | p.His308Glnfs*16 | 6 | *USH2A* seq | - | - | Dreyer et al 2000, Dreyer et al 2008 |
| USH2-50 (D23) | M | 7-8 yrs | NA | + | + | c.2299delG | p.Glu767Serfs*21 | 13 | c.1876C>T | p.Arg626* | 11 | *USH2A* seq | - | - | Dreyer et al 2000, Dreyer et al 2008 |
| USH2-51 (D30) | F | Always | NA | - | + | c.2299delG | p.Glu767Serfs*21 | 13 | c.5473G>T | p.Glu1825* | 27 | *USH2A* seq | - | - | Dreyer et al 2000, Dreyer et al 2008 |
| USH2-52 | F | NA | NA | - | + | c.2299delG | p.Glu767Serfs*21 | 13 | c.3407G>A | p.Ser1136Asn | 17 | *USH2A* seq | - | - | Present study |
| USH2-52a | M | NA | NA | NA | NA | c.2299delG | p.Glu767Serfs*21 | 13 | c.3407G>A | p.Ser1136Asn | 17 | *USH2A* seq | - | - |  |
| USH2-53 (D31) | F | 18 yrs | NA | + | + | c.2299delG | p.Glu767Serfs*21 | 13 | c.10345delinsAA | p.Glu3449Lysfs*25 | 52 | *USH2A* seq | - | - | Dreyer et al 2000, Dreyer et al 2008 |
| USH2-54 (D49) | M | NA | NA | - | NA | c.2299delG | p.Glu767Serfs*21 | 13 | c.10510C>A, | p.Pro3504Thr | 53 | *USH2A* seq | - | - | Dreyer et al 2000, Dreyer et al 2008 |
| USH2-55 (D04) | F | Always | NA | + | + | c.2299delG | p.Glu767Serfs*21 | 13 | c.2299delG | p.Glu767Serfs*21 | 13 | *USH2A* seq | - | - | Dreyer et al 2000, Dreyer et al 2008 |
| USH2-56 (D09) | F | 17-18 yrs | NA | + | + | c.2276G>T | p.Cys759Phe | 13 | c.5653A>T | p.Arg1885* | 28 | *USH2A* seq | - | - | Dreyer et al 2000, Dreyer et al 2008 |
| USH2-57 (D15) | M | 11 yrs | + | + | + | c.2299delG | p.Glu767Serfs*21 | 13 | c.920_923dup | p.His308Glnfs*16 | 6 | *USH2A* seq | - | - | Dreyer et al 2000, Dreyer et al 2008 |
| USH2-58 (D12) | M | 16 yrs | NA | + | + | c.1965delT | p.Cys655Trpfs*101 | 11 | c.11864G>A | p.Trp3955* | 61 | *USH2A* seq | - | - | Dreyer et al 2000, Dreyer et al 2008 |
| USH2-59 | F | 18 yrs | + | + | + | c.2299delG | p.Glu767Serfs*21 | 13 | c.2299delG | p.Glu767Serfs*21 | 13 | *USH2A* seq | - | - | Present study |
| USH2-60 (D56) | M | 20 yrs | - | + | + | c.5270A>G; c.9370A>G | p.Tyr1757Cys; p.Arg3124Gly | 26, 47 | c.10684G>T | p.Glu3562* | 54 | *USH2A* seq | - | - | Dreyer et al 2000, Dreyer et al 2008 |
| USH2-61 (D27) | F | 29 yrs | NA | + | + | c.2299delG | p.Glu767Serfs*21 | 13 | c.14384T>G | p.Leu4795Arg | 66 | *USH2A* seq | - | - | Dreyer et al 2000, Dreyer et al 2008 |
| USH2-62 (D59) | F | NA | NA | + | + | c.2299delG | p.Glu767Serfs*21 | 13 | NI | NI |  | *USH2A* seq | - | - | Dreyer et al 2000, Dreyer et al 2008 |
| USH2-63 | M | NA | NA | + | NA | c.2299delG | p.Glu767Serfs*21 | 13 | NI | NI |  | APEX | - | - | Tranebjaerg et al 2011 |
| USH2-64 | F | 10-11 yrs | - | + | + | c.2299delG | p.Glu767Serfs*21 | 13 | c.7195_7207del | p.Ile2399Phefs*10 |  | APEX/*USH2A* seq | - | - | Tranebjaerg et al 2011 |
| USH2-65 | F | 13 yrs | NA | + | + | c.2023C>T | p.Gln675* | 12 | c. 2023C>T | p.Gln675* | 12 | APEX | - | - | Dreyer et al 2000, Dreyer et al 2008 |
| USH2-66 | F | 30 yrs | NA | - | + | c.2299delG | p.Glu767Serfs*21 | 13 | NI | NI |  | APEX/*USH2A* seq | - | - | Tranebjaerg et al 2011 |
| USH2-68 (D11) | F | NA | NA | + | NA | c.2299delG | p.Glu767Serfs*21 | 13 | c.2299delG | p.Glu767Serfs*21 | 13 | *USH2A* seq | - | - | Dreyer et al 2000, Dreyer et al 2008 |
| USH2-74 | M | NA | NA | NA | + | NI | NI |  | NI | NI |  | Targeted NGS of USH genes (Otogenetics) Ot9088 | - | - | Present study |
| USH2-77 | F | NA | NA | NA | NA | NI | NI |  | NI | NI |  | Targeted NGS of USH genes (Otogenetics) Ota2312. | - | - | Present study |
