# Supplementary material for: Usher syndrome in Denmark: mutation spectrum and some clinical observations
Source: Mol Genet Genomic Med. 2016 Jun 28;4(5):527–39. doi: 10.1002/mgg3.228 (PMC5023938; doi:10.1002/mgg3.228)
Supplement: Supplementary file 3 — Table S3. Clinical data and mutations identified in an individual with USH3. −: absent, +: present; mutations in bold: novel according to HGMDprof and LOVD USH database 161015; Accession number: CLRN1 (NM_174878.2). [file MGG3-4-527-s003.docx]

| **Patient** | **Gender** | **Onset of night blindness** | **Cataract** | **Macula edma** | **Audiologica: early onset of progressive HI (USH3)** | **Gene** | **Allele 1** | **Allel 1 (predicted effect)** | **Exon** | **Allele 2** | **Allel 2 (predicted effect)** | **Exon** | **Method** | **Origin** | **Consanguinity** | **Remarks** | **Patient published** |
| --- | --- | --- | --- | --- | --- | --- | --- | --- | --- | --- | --- | --- | --- | --- | --- | --- | --- |
| USH3-1 | F | always nightblind | + | - | + | *CLRN1* | **c.254-1G>A; IVS1-1G>A** | p.? |  | **c.254-1G>A; IVS1-1G>A** | p.? |  | *CLRN1* sequencing | Syria | + | - | Present study |
