# Supplementary material for: Usher syndrome in Denmark: mutation spectrum and some clinical observations
Source: Mol Genet Genomic Med. 2016 Jun 28;4(5):527–39. doi: 10.1002/mgg3.228 (PMC5023938; doi:10.1002/mgg3.228)
Supplement: Supplementary file 4 — Table S4. Primer list and PCR program. Table includes all primers used for verification of the variants identified by NGS and primers for Sanger sequencing of the intronic variant c.7595‐2144A>G in USH2A. [file MGG3-4-527-s004.docx]

**Supplementary Table 4**: List of primers

| **Gene** | **Mutation** | **Primer name** | **Sequence** |
| --- | --- | --- | --- |
| **Primer used for Sanger sequencing in order to verify variants identified by NGS** | | | |
| *USH2A* | p.Arg3124Gly | c.9370A>G_ L | TGGCTCAAAAACCAAGAAAA |
| *USH2A* | p.Arg3124Gly | c.9370A>G_ R | GAATTCAAGCGTGCACCAC |
| *CDH23* | p.Gln1288* | c.3862C>T_L | TGCCATCCACATTGACCTTA |
| *CDH23* | p.Gln1288* | c.3862C>T_R | CTTGGCCATCTCAAGGACAT |
| *CDH23* | p.Glu2173* | c.6517G>T_ L | CTCCCTTTTCCCTCTCCAAC |
| *CDH23* | p.Glu2173* | c.6517G>T_ R | GAGAAAGAAGGGGGAAGCAG |
| *MYO7A* | p.Arg1168Gln | c.3503G>A_ L | GTGGCAGGGGTAATGACAGT |
| *MYO7A* | p.Arg1168Gln | c.3503G>A_ R | GCAATATCTCCAAGGGATGC |
| *USH2A* | p.Glu767Serfs*21 | c.2299delG_L | GCAAGGCAGACAGAGGAAAG |
| *USH2A* | p.Glu767Serfs*21 | c.2299delG_R | TGGCATTGCTTGTGAGAAAA |
| *USH2A* | p.Cys536Arg | c.1606T>C_ L | CCTTAGGGTTTGGTTTCAGTTG |
| *USH2A* | p.Cys536Arg | c.1606T>C_ R | ACGGGTTTGGATATTGGTGA |
| *MYO7A* | p.Lys269del | c.805_807del_ L | TTCCCTCTGTGCTGGAAATC |
| *MYO7A* | p.Lys269del | c.805_807del_ G | TGGCTAAAGGAGTGGTGCTT |
| *MYO7A* | p.Arg570* | c.1708C>T_L | CTTCTTGAACAGGCCTGGAA |
| *MYO7A* | p.Arg570* | c.1708C>T_R | ACAAGGGCAGGGCTTAGACT |
| *MYO7A* | p.Cys31* | c.93C>A_ L | AAATGGGAATATGGCTGTTCT |
| *MYO7A* | p.Cys31* | c.93C>A_ R | CCACCACACACACACACATC |
| *USH2A* | p.Leu4795Arg | c.14384T>G_ L | CTGCCTGACCCATTACCTGT |
| *USH2A* | p.Leu4795Arg | c.14384T>G_ R | ATGCCTTGTGATTTCCATCC |
| *USH2A* | p.Trp3521Arg | c.10561T>C_ L | TGCCTTGCTTGTTTTGAAAG |
| *USH2A* | p.Trp3521Arg | c.10561T>C_R | TGAAAATAAGAGAAACTTCCCTGTC |
| *USH2A* | c.486-14G>A | c.486-14G>A_L | TAAAGGGGCTTCTTCATTGG |
| *USH2A* | c.486-14G>A | c.486-14G>A_R | TTGGGGGAGGAAATGCTAGT |
| **Primers used for Sanger sequencing of the intronic variant** | | | |
| *USH2A* | c.7595-2144A>G | c.7595-2144A>G_L | GGTTTTCATCTGGGTCTTGC |
| *USH2A* | c.7595-2144A>G | c.7595-2144A>G_R | TCCCTAGCCAAAGGAGCTAA |

PCR amplifications for all listed primer pairs were performed with the following cycling parameters: 15 min at 95°C; 40 cycles of 94°C for 1 min, 60°C for 1 min and 72°C for 1 min; 7 min final extension at 72°C.
